# Laboratory Findings and Clinical Outcomes of ICU-admitted COVID-19 Patients: A Retrospective Assessment of Particularities Identified among Romanian Minorities

**DOI:** 10.3390/jpm13020195

**Published:** 2023-01-21

**Authors:** Alexandra Mocanu, Voichita Elena Lazureanu, Ruxandra Laza, Adelina Raluca Marinescu, Talida Georgiana Cut, Suzana-Vasilica Sincaru, Adina Maria Marza, Irina-Maria Popescu, Lucian-Flavius Herlo, Andreea Nelson-Twakor, Mircea Rivis, Felix Bratosinand, Tamara Mirela Porosnicu, Alexandru Ovidiu Mederle

**Affiliations:** 1 Department XIII, Discipline of Infectious Diseases, “Victor Babes” University of Medicine and Pharmacy, 300041 Timișoara, Romania; 2 Emergency Institute for Cardiovascular Diseases and Transplantation, Strada Gheorghe Maricescu, 540327 Targu Mures, Romania; 3 Department of Surgery, Multidisciplinary Center for Research, Evaluation, Diagnosis and Therapies in Oral Medicine, “Victor Babes” University of Medicine and Pharmacy Timisoara, Eftimie Murgu Square 2, 300041 Timisoara, Romania; 4 Department XIII, Discipline of Epidemiology, “Victor Babes” University of Medicine and Pharmacy Timisoara, Eftimie Murgu Square 2, 300041 Timisoara, Romania; 5 Department of Surgery, Ineu City Hospital, Republicii Street 2, 315300 Arad, Romania; 6 Faculty of Medicine and Pharmacy, “Ovidius” University of Constanta, 900527 Constanta, Romania; 7 Department of Anesthesiology and Oral Surgery, Multidisciplinary Center for Research, Evaluation, Diagnosis and Therapies in Oral Medicine, “Victor Babes” University of Medicine and Pharmacy Timisoara, Eftimie Murgu Square 2, 300041 Timisoara, Romania; 8 Intensive Care Unit, “Victor Babes” Hospital for Infectious Disease and Pneumology, Strada Gheorghe Adam 13, 300041 Timisoara, Romania

**Keywords:** COVID-19, SARS-CoV-2, Roma population, intensive care, inflammatory markers

## Abstract

The Roma population accounts for over 3% (approximately 10 to 15 million) of Romania’s permanent population, and it represents one of Europe’s most impoverished populations. Due to poverty and unemployment, Romania’s Roma minority may have diminished access to healthcare and preventive medicine. The limited existing evidence suggests that the European Roma group has been at a higher risk of becoming ill and dying during the pandemic owing to their lifestyle choices, socioeconomic circumstances, and genetic pathophysiological traits. As a result, the purpose of the present research was to investigate the link between the inflammatory markers implicated and the clinical progression of COVID-19 in Roma patients who were brought to the intensive care unit. We considered 71 Roma patients admitted to the ICU with SARS-CoV-2 infection and 213 controls from the general population with the same inclusion criteria. The body mass index of patients was statistically significantly higher among Roma patients, with more than 57% being overweight, compared with 40.7% in the control group. Frequent smoking was more prevalent in patients of Roma ethnicity admitted to the ICU and the number of comorbidities. We observed a significantly higher proportion of severe imaging features at admission in the group of cases, although this difference may have been associated with the higher prevalence of smoking in this group. The mean duration of hospitalization was longer by 1.8 days than the control group. Elevated ESR levels were observed in 54.0% of Roma patients at admission, compared with 38.9% in the control group. Similarly, 47.6% of them had elevated CRP levels. IL-6 increased significantly at the time of ICU admission, similarly to the significant rise in the CRP levels, compared with the general population. However, the proportion of intubated patients and mortality did not differ significantly. On multivariate analysis, the Roma ethnicity significantly influenced the CRP (β = 1.93, *p*-value = 0.020) and IL-6 (β = 1.85, *p*-value = 0.044). It is necessary to plan different healthcare strategies aimed at special populations, such as the Roma ethnicity, to prevent the reduced disparities presented in in this study.

## 1. Introduction

In the majority of patients, SARS-CoV-2 causes no symptoms or only mild symptoms; it is less fatal than other viral infections, even though up to 20% of cases, such as those involving older people and those with multiple comorbidities, may develop severe forms and overactivation of the immune system [1,2,3]. The most common symptoms of COVID-19 are generally nonspecific, including high prevalence rates of fever, tiredness, and a dry cough. On the other hand, interstitial pneumonia, thrombo-embolic events, and acute respiratory distress syndrome (ARDS) are all outcomes and possible severe manifestations of SARS-CoV-2 infection among populations at risk [4,5,6,7,8]. These forms of COVID-19 are likely to be triggered by an overactivation of the immune system, causing a cytokine storm [9,10,11,12].

The excessive inflammation seen in certain patients, particularly those who develop severe disease, is one of the most distinctive characteristics of COVID-19. An overactive immune response that is driven by several different cytokines is one factor that contributes to the development of severe illness [13]. In the research conducted so far, some immune cells and inflammatory mediators have been identified as being involved in the illness process. These markers include lymphokines, cytokines, monokines, tumor necrosis factors (TNF), and interferons, all with autocrine, paracrine, or endocrine effects [14,15]. In order to battle the potentially lethal inflammation, researchers from all over the globe have investigated a wide variety of pharmacotherapeutic medicines, including anti-inflammatory drugs and antivirals, although without any breakthrough discovery in COVID-19 treatment [16,17].

The cytokine storm syndrome is one of the most severe complications that can occur in COVID-19 patients, being triggered by the inflammatory cell infiltration in the lungs, activation of T-helper 1 reactions, and abundant release of proinflammatory cytokines into the circulation [18,19]. The systemic inflammation can cause multiple organ dysfunction syndromes (MODS) and disseminated intravascular coagulation (DIC). Therefore, the prompt treatment of this cytokine storm in its early stage, with the use of immunomodulators, corticosteroids, and cytokine antagonists, is suggested by some specialists to be the most important factor in lowering the death rate of these individuals and determining fewer admissions to intensive care units [20] This treatment can be accomplished by administering these medications when the cytokine storm is in its early stage.

Many COVID-19 patients who exhibit severe SARS-CoV-2 infection are admitted to critical care units and have very high concentrations of inflammatory markers and D-dimers in their serum samples [21]. When evaluating the results of blood tests on patients with severe SARS-CoV-2 infection, it was observed that a very large increase in cytokines, roughly equivalent to an increase of four times or more, is associated with an increased risk of mortality [22]. Additionally, the severity of COVID-19 may be impacted by a number of known and unknown variables, such as certain demographic features, racial disparities, the poverty status of the community, or an ethnic group such as the Roma population. 

The findings of a few studies indicate that people of Roma ethnicity, also known as Romani in the European Union or Gypsies as a pejorative form, are likely to be at an increased risk of SARS-CoV-2 infection [23]. In addition, the Roma community is likely to suffer psychological, social, and economic repercussions directly from the pandemic. In addition, it seems that some racial and ethnic groups are more prone to comorbidities that predispose them to poorer COVID-19 results, as well as certain unique genetic characteristics that relate the severity of infection with the demographic profile of those groups. Similarly, it is expected that Roma patients are less likely to be vaccinated against COVID-19; therefore, they might have a more severe response to the viral infection [24,25,26]. To the best of our knowledge, however, there is a paucity of data about the dynamics of SARS-CoV-2 viral manifestations in the Roma population. As a consequence, the objective of this study was to explore the clinical development of COVID-19 in Roma patients concerning the laboratory findings and inflammatory markers that were involved. 

## 2. Materials and Methods

### 2.1. Study Design and Ethics

Patients were eligible for participation in the present observational retrospective research provided it was determined that their hospital admission occurred between March 2020 and August 2022. This timeframe encompasses both the pre-COVID-19 vaccination phase and the post-COVID-19 vaccine period. The investigation was conducted at the “Victor Babes” University of Medicine and Pharmacy in Timisoara. More specifically, it took place in the “Victor Babes” Hospital for Infectious Disease and Pneumology under the Department of Infectious Disease. The purpose of this study was to conduct research in retrospect by collecting data from the paper records and digital records of patients diagnosed with COVID-19 who were admitted to the hospital throughout the time period of the study.

As an auxiliary of the “Victor Babes” Hospital for Infectious Disease and Pneumology in Timisoara, the infectious disease clinic affiliated with the “Victor Babes” University of Medicine and Pharmacy operates under the laws of the local commission of ethics that approves scientific research that operates following the International Conference on Harmonization from Helsinki regarding technical requirements for registration of pharmaceuticals for human use. Additionally, the infectious disease clinic is governed by the local commission of ethics laws that approve scientific studies. The research was conducted in a manner that accorded with the ethical standards of the university at which the study was conceived, as well as by the ethics committees of both institutions.

### 2.2. Inclusion Criteria and Variables

A database and patient paper record search were conducted to determine the number of adult Roma patients admitted to the ICU with a COVID-19 diagnosis. Patients were included if they matched the following criteria: (1) being older than 18 years; (2) if their paper records mentioned the Roma minority; (3) being vaccinated or unvaccinated against SARS-CoV-2; (4) having a SARS-CoV-2 infection confirmed by PCR test. According to existing guidelines, the SARS-CoV-2 infection was considered mild, moderate, or severe [27,28]. Severe imaging features were considered for ground-glass opacities involving more than 50% of the lungs on chest X-ray or CT scan [6]. The COVID-19 status was defined by a positive polymerase chain reaction test (PCR) from oropharyngeal and nasal swabs, using a multiplex RT-PCR [29,30]. A predefined patient personal form was used to gather demographic, clinical, and outcome data from electronic medical records and identify the patients’ ethnicity. 

Considering that, after January 2021, the COVID-19 vaccination campaign started in Romania, it was considered necessary to stratify the cohort of patients into vaccinated Roma, unvaccinated Roma, and a control group from the general population of patients with SARS-CoV-2 infection, excluding those of Roma ethnicity. Vaccination status was identified with the unique QR code of the European Union COVID-19 vaccination certificate. Patients were vaccinated with the available vaccines in Romania produced by Pfizer/BioNTech (Reinbek, Germany), AstraZeneca (Oxford, UK), Moderna (Norwood, MA, USA), and Johnson & Johnson (New Brunswick, NJ, USA).

Using a convenience sample method, we estimated that at least 27 patients of Roma minority should be included in the analysis to provide proper statistical power. The sample size was calculated for a proportion of Roma ethnicity in Romania of about 3.5%, according to the most recent census [31], and the rate of ICU admission of about one-third of the severe cases [32]. Other considerations for the sample size were a 99% confidence level and a 5% margin of error. A total of 71 patients from the Roma population that matched the inclusion criteria were included in the analysis. The control group from the general population was case-matched by age and COVID-19 vaccination status with the Roma group and included 213 patients. 

The variables taken into consideration included background data (age, gender, area of residence, occupation, body mass index, smoking status, and alcohol use), the presence of chronic comorbidities (high blood pressure, lung disease, diabetes mellitus, cerebrovascular disease, digestive and liver problems, kidney disease, depression, and malignancy), and COVID-19 transmission source. COVID-19 data that were analyzed comprised signs and symptoms, COVID-19 patient outcomes, and COVID-19 treatment. Clinical presentation of the patients comprised their oxygen saturation on admission, respiratory rate, and heart rate. According to the existing national guidelines [33], clinical picture, and the number of comorbidities, COVID-19 patients received antiviral agents, broad-spectrum antibiotics, anticoagulant treatment, steroids, and immune modulators for the duration of hospital admission. Lastly, the laboratory data included the following inflammatory markers: procalcitonin, D-dimers, IL-6, TNF-alpha, ferritin, ESR, CRP, and fibrinogen.

### 2.3. Statistical Analysis

IBM SPSS v.27(IBM, Armonk, New York, USA) and MedCalc v.20 (MedCalc Software Ltd., Ostend, Belgium) were used for statistical analysis. We calculated the absolute (n) and relative (%) frequencies of categorical variables and compared their proportions using chi-square and Fisher’s exact test. After testing the available data for normality with the Shapiro–Wilk test, we used the Mann–Whitney test to compare non-Gaussian variables, and we reported them by the median and interquartile range (IQR). The mean and standard deviation of continuous variables with a normal distribution were compared using the Student’s *t*-test (unpaired, independent samples). A Kaplan–Meier probability plot was created to estimate mortality risk. A multivariate regression analysis was performed to determine the influence of patients’ ethnicity for elevated inflammatory markers at hospital admission. A significance level of 0.05 was chosen as the alpha value.

## 3. Results

### Patients’ Background Characteristics

Table 1 presents a comparison of baseline characteristics between cases (patients of Roma ethnicity) and controls from the general population that were diagnosed with COVID-19 and required admission to the ICU. A total of 71 cases were included in the analysis, and 213 controls with a 1:3 ratio and case-matched by age and COVID-19 vaccination status. The majority of patients were at retirement age, over 65 years old (>47%), being represented more often by the male gender (>54%). The body mass index of patients was statistically significantly higher among Roma patients, with more than 57% being overweight, compared with 40.7% in the control group. 

Other background characteristics of the study participants identified a higher prevalence of Roma patients residing in the rural regions of Romania, with significantly more of them being unemployed (38.0% vs. 25.8%, *p*-value = 0.049). Frequent smoking was also more prevalent in patients of Roma ethnicity (38.0% vs. 24.9%, *p*-value = 0.032). Another important finding was that Roma patients admitted to the ICU had more comorbidities, with 53.5% having three or more comorbid conditions, compared with 34.7% in the control group (*p*-value = 0.017). Moreover, 12.7% of cases vs. 12.2% of controls were immunized with COVID-19 vaccines. 

Table 2 describes the SARS-CoV-2 infection signs, symptoms, and outcomes in Roma patients and the control group of patients admitted to the ICU. There were no statistically significant differences between the signs and symptoms of the studied patients and no differences in the COVID-19 treatment. However, we observed a significantly higher proportion of severe imaging features at admission in the group of cases (40.8% vs. 28.2%, *p*-value = 0.046), although this difference can be associated with the higher prevalence of smoking in the same group.

Data in Table 3 describe the comparison of COVID-19 outcomes between patients of cases and controls admitted to the ICU. It was observed that the mean duration of hospitalization was longer by 1.8 days than the control group (*p*-value = 0.027). The viral clearance had a significantly longer duration in the Roma ethnicity group, confirming the longer mean duration of hospitalization. The median duration from symptom onset to hospital admission was 1 day shorter in the group of Roma patients, although not statistically significant. Similarly, the median duration from hospital admission to ICU admission was also shorter in the cases group (*p*-value = 0.113). The SOFA score and proportion of severe in-hospital complications did not show significant differences between the study groups. However, the median duration of ICU stay was significantly longer in the Roma group (7 days vs. 5 days, *p*-value < 0.001), and the proportion of intubated patients and mortality did not differ significantly.

When examining the cytokines and inflammatory markers at hospital admission between cases and controls, it was observed that ESR and CRP levels were significantly increased in the Roma patient group, as presented in Table 4. Thus, elevated ESR levels were observed in 54.0% of Roma patients at admission, compared with 38.9% in the control group. The median ESR levels in the cases group was 26 mm/h, compared to 20 mm/h in the control group (*p*-value = 0.002). Similarly, 47.6% patients from the cases group had elevated CRP levels, with a median value of 24 mg/dL, compared with 17 mg/dL among controls, (*p*-value < 0.001).

**Figure 1:**
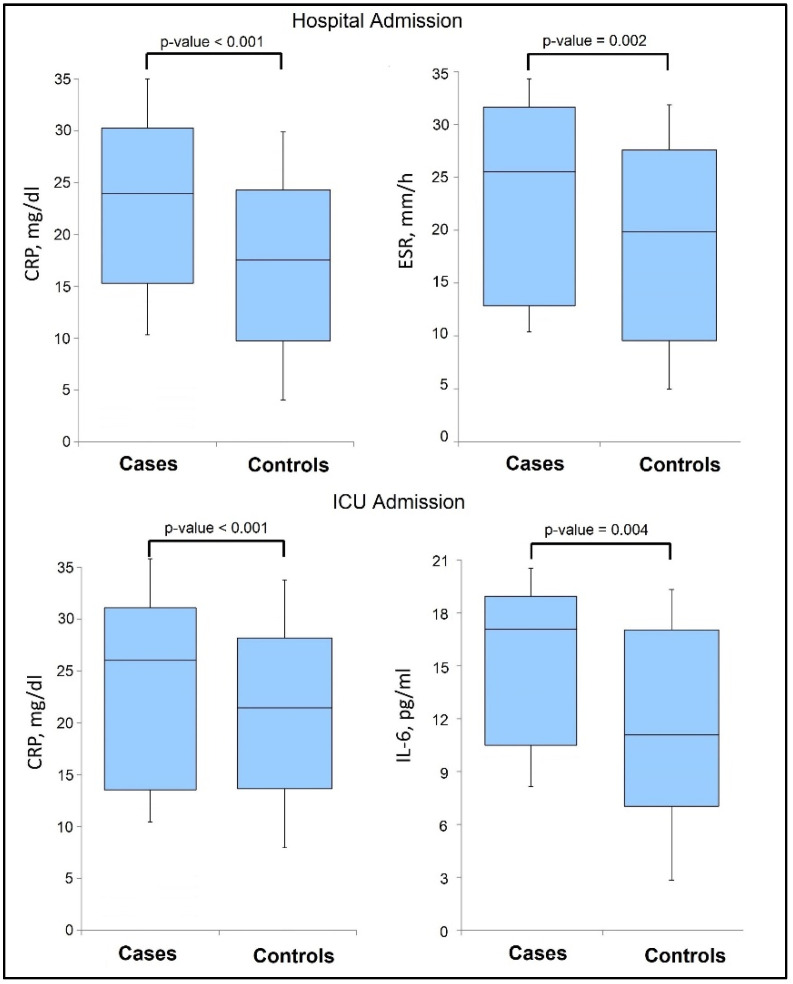
Boxplot of significant findings in biological parameters at hospital admission and ICU admission. The upper boxes present the median and IQR values of CRP and ESR at hospital admission. The lower boxes present the median and IQR values of CRP and IL-6 at ICU admission.

**Figure 2:**
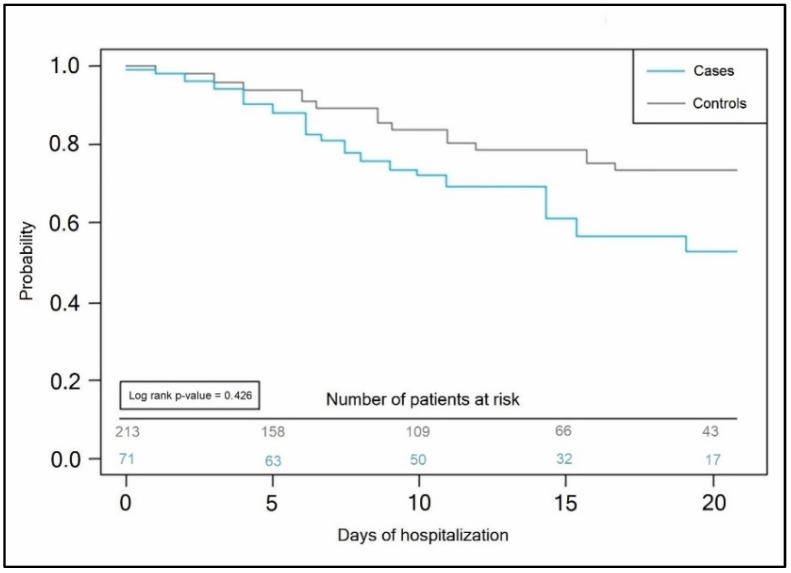
Kaplan–Meier probability curve of survival after ICU admission between patients of Roma ethnicity and the general population.

It was observed that, at the moment of ICU admission, the inflammatory markers worsened, increasing in a majority of patients, as seen in Table 5, as compared to the findings observed at admission. IL-6 levels increased significantly in both groups, although with a statistically significant difference between the cases and control groups (17 pg/mL vs. 11 pg/mL, *p*-value = 0.004). CRP levels that were increased since admission also remained significantly different between the two study groups, with 62.0% of Roma ethnicity patients having elevated CRP at ICU admission, compared with 46.0% in the general population group. The median CRP value among Roma patients was 27 mg/dL, compared with 21 mg/dL (*p*-value < 0.001), as seen in Figure 1. Lastly, the Kaplan–Meyer probability curve of mortality after ICU admission between patients of Roma ethnicity and the general population showed similar risks in both groups, with no significant differences (log-rank *p*-value = 0.426), as presented in Figure 2.

A multivariate regression analysis was performed to determine the influence of patients’ ethnicity for elevated inflammatory markers at hospital admission, presented in Table 6. The control group from the general population was considered as the reference group for risk analysis, while CRP, ESR, and IL-6 were considered the dependent variables potentially influenced by the ethnicity of the patients. Only these inflammatory markers were considered for inclusion in the regression analysis after previously determining a statistically significant difference between the cases and control groups regarding these three serum markers. The regression was performed using a threshold for the dependent variables as the upper value of the normal range, and two times the upper value of the normal range. It was observed that the general population did not influence significantly the variation of inflammatory markers above the normal range or two times above the normal range. On the other hand, Roma ethnicity did not have a significant influence on these markers elevated one time above the normal range; however, at two times the normal range, it was shown that it influenced significantly the CRP (β = 1.93, *p*-value = 0.020) and IL-6 (β = 1.85, *p*-value = 0.044).

## 4. Discussion

### 4.1. Literature Findings

The current study identified several particularities that seem to affect more often or more severely the COVID-19 patients of Roma ethnicity. One of the minority groups that are the most disadvantaged and underdeveloped on the European continent is the Roma ethnicity [34]. Roma communities have drastically worse health outcomes, such as a far shorter life expectancy, an increased prevalence of both physical and mental health concerns, and larger adoption of risky health practices. Roma communities also have much higher rates of substance abuse, as observed in our study. Contagious diseases, including measles, hepatitis, and TB, disproportionately impact Roma communities due to their lifestyle pattern of living in close groups with many family members [35]. In addition, Roma ethnics are less likely to participate in healthcare services, including their reluctance to vaccination programs, child health, and maternal care, due to barriers such as culture, language, and health literacy, making it less likely for this minority to receive primary and preventive care [36].

According to the results of our research, the unemployment rate among Roma patients was much higher than the general population, and the majority of them lived in rural areas. In addition to these factors, the lifestyle conditions previously described that characterize people of Roma ethnicity might disproportionately expose them to higher risks of SARS-CoV-2 infection, as well as the risk of other contagious diseases due to a higher risk of viral transmission within their communities. It is also more difficult for disadvantaged groups to engage in mitigation activities such as frequently washing their hands, keeping a physical distance, and receiving access to medical care [37]. Therefore, the increased hazards presented by COVID-19 for these communities have the potential to make the health disparities that now exist among Roma groups considerably worse while also potentially having a negative impact on the health of the broader population. 

Although no other researchers have reported similar particular results addressing the inflammatory markers in minorities, such as the Roma ethnicity, IL-6 levels have been linked to prolonged lung damage in SARS-CoV-2-infected individuals [38,39]. Notably, these studies described how individuals with persistent pulmonary lesions, as determined by high CT scores, had elevated IL-6 levels at discharge and during the follow-up period, which may also explain why our group of patients with higher IL-6 at admission had more ICU admissions and oxygen supplementation requirements [40]. Importantly, the peak expression of IL-6 prior to the worsening of lung damage was mostly seen in patients with persistent lesions, and multivariate analysis demonstrated that the IL-6 level at admission was an independent predictor of persistent pulmonary injury [41]. Consequently, this much greater percentage of Roma patients with severe COVID-19 who had increased IL-6 levels at admission might explain why these patients also had persistently elevated inflammatory markers, such as CRP and ESR, upon admission.

The elevated CRP levels seen in individuals with severe SARS-CoV-2 infection may be attributed to the overproduction of inflammatory cytokines. When the immune system is overactive, cytokines may cause irreparable lung tissue damage. Therefore, the production of CRP is enhanced by both inflammatory cytokines and the degradation of tissue in COVID-19-infected patients [42]. In conclusion, an elevated CRP level may be a valuable early marker for predicting the likelihood of disease development in COVID-19 patients with mild symptoms. This may allow medical personnel to identify such individuals at an early stage in order to initiate early treatment. In addition, COVID-19 patients with elevated CRP levels need continual monitoring and treatment, even if they did not develop symptoms consistent with a severe disease course [43]. In more than 70% of the COVID-19 intubated patients from the Roma ethnicity, there was a significant increase in C-reactive protein levels. Moreover, severe cases of SARS-CoV-2 infection had higher C-reactive protein levels compared to moderate disease patients in most of the studies [44].

We observed that Roma patients were admitted to the ICU within 3.5 days from the initial hospitalization and 7 days from the onset of their symptoms, which is significantly lower than the general population. This length was, on average, 1 day shorter than the overall population, although this difference was not statistically significant, probably due to a much greater prevalence of comorbidities, such as diabetes mellitus, hypertension, heart disease, and dyslipidemia, among ethnic Roma. In this cohort study, patients of Roma ethnicity were observed to have several elevated inflammatory biomarkers that were significantly higher than in the general population, such as CRP, ESR, and IL-6. Certain inflammatory markers were significantly greater in Roma patients and were related to higher rates of ICU admission or illness severity, but a causative relationship could not be established. In addition, these individuals had a greater prevalence of comorbidities, such as obesity, which is linked with a more severe systemic inflammatory status. It can be hypothesized that these elements, together with a possible genetic predisposition, define greater risks. Although Roma patients had a higher mortality rate, the difference was not statistically significant. There is a good chance that stringent medical and therapeutic care in the ICU saved many lives and decreased the death rate in this demographic. Viral clearance and duration of hospitalization were significantly longer in the Roma population, likely correlated with the more elevated inflammatory markers. 

The same Roma patients also had higher median SOFA scores upon admission to the ICU; however, the difference was not statistically significant. Similar studies reported the SOFA score as a strong predictor of mortality in patients with severe COVID-19 [45,46]. Other good predictors for mortality in SARS-CoV-2 infections were the increased circulating cytokines, mostly interleukins. However, one meta-analysis observed that people of Asian descent had lower levels of interleukins when compared to the European population [47], thus confirming that patients of Roma ethnicity might exhibit different pathophysiology during COVID-19, although future populational studies are recommended.

### 4.2. Study Limitations

Even though the selected cohort met the criteria for the sample size needed to achieve statistical power, the number of patients was still not large enough to find COVID-19 outcomes with a lower incidence. In a similar manner, the sample size might have affected the results found in this study, considering that Roma patients were admitted with significantly more elevated inflammatory markers, but the mortality rate was not significantly higher. Nevertheless, the supportive treatment received in the ICU could also help in lowering the mortality odds. Since all of the patients in this study were admitted to a tertiary hospital, it is important to point out the potential for bias in the patient selection that exists within this research, having a higher likelihood of admitting more severe cases requiring ICU admission. As a result, it is possible that the instances of COVID-19 that have been described might have a severity that is higher than the norm.

## 5. Conclusions

We believe that patients of Roma ethnicity exhibit particular population-specific features that manifest differently when facing a disease such as COVID-19. This study showed that Roma patients admitted to the ICU did not have more frequent first symptoms than the general population, such as fever, shortness of breath, and cough. However, they had more risk factors for mortality after intubation, which is likely to be influenced by a higher proportion of comorbid conditions and unhealthy behavior such as smoking, in this particular population. Some inflammatory markers such as ESR, CRP, and IL-6 were significantly more elevated in some of the cases, which could potentially increase the mortality rates. However, in reality, we did not observe a significantly higher rate of Roma patients being intubated or a higher mortality, which can be attributed to good, individualized treatment and management in the ICU. Additional prospective studies must be conducted in order to address more specific laboratory markers of the infected individuals that are highly correlated with a severe SARS-CoV-2 infection and to find the most effective therapy methods.

## Figures and Tables

**Table 1:** Comparison of baseline characteristics between cases and controls with COVID-19 admitted to the ICU.

Variables *	Cases (*n* = 71)	Controls (*n* = 213)	*p*-Value
Background Data			
Age			0.997
18–40 years	13 (18.3%)	39 (18.3%)	
40–65 years	24 (33.8%)	73 (34.3%)	
>65 years	34 (47.9%)	101 (47.4%)	
Sex			0.448
Men	42 (59.2%)	115 (54.0%)	
Women	29 (40.8%)	98 (46.0%)	
BMI			0.043
Underweight (<18.5 kg/m^2^)	4 (4.8%)	14 (5.1%)	
Normal weight (18.5–25.0 kg/m^2^)	27 (37.3%)	1115 (54.2%)	
Overweight (>25.0 kg/m^2^)	40 (57.8%)	84 (40.7%)	
Other characteristics			
Area of residence (urban)	32 (45.1%)	132 (62.0%)	0.012
Occupation (unemployed)	27 (38.0%)	55 (25.8%)	0.049
Relationship status (married)	66 (93.0%)	194 (91.1%)	0.622
Substance use behavior			
Chronic smoking	27 (38.0%)	53 (24.9%)	0.032
Chronic alcohol use	6 (8.5%)	14 (6.6%)	0.592
Number of comorbidities			0.017
0	7 (9.9%)	36 (16.9%)	
1	12 (16.9%)	31 (14.6%)	
2	14 (19.7%)	72 (33.8%)	
≥3	38 (53.5%)	74 (34.7%)	
Wave of SARS-CoV-2 infection			0.506
1	13 (18.3%)	33 (15.5%)	
2	16 (22.5%)	41 (19.2%)	
3	8 (11.3%)	15 (7.0%)	
4	9 (12.7%)	22 (10.3%)	
5	18 (25.4%)	66 (31.0%)	
6	7 (9.9%)	36 (16.9%)	
COVID-19 vaccination status			0.916
Yes	9 (12.7%)	26 (12.2%)	
No	62 (87.3%)	187 (87.8%)	
COVID-19 vaccine	(*n* = 9)	(*n* = 26)	0.757
BNT162b2	3 (33.3%)	11 (42.3%)	
mRNA-1273	2 (22.2%)	7 (26.9%)	
Ad26.COV2.S	4 (44.4%)	8 (30.8%)	
COVID-19 transmission source			0.882
Community	11 (15.5%)	38 (17.8%)	
Family	27 (38.0%)	76 (35.7%)	
Unknown source	33 (46.5%)	99 (46.5%)	

* Data reported as n (%) and calculated using Chi-square test and Fisher’s exact unless specified differently. BMI—body mass index; BNT162b2—Pfizer/BioNTech; mRNA-1273—Moderna; Ad26.COV2.S—Johnson & Johnson.

**Table 2:** Comparison of SARS-CoV-2 infection signs, symptoms, and outcomes between patients of cases and controls admitted to the ICU.

Variables *	Cases (*n* = 71)	Controls (*n* = 213)	*p*-Value
Signs and Symptoms			
Cough	52 (62.7%)	160 (67.8%)	0.393
Fever	59 (71.1%)	173 (73.3%)	0.695
Dyspnea	43 (51.8%)	127 (53.8%)	0.752
Headache	10 (12.0%)	38 (16.1%)	0.374
Digestive symptoms	21 (25.3%)	43 (18.2%)	0.165
Anosmia/ageusia	24 (28.9%)	71 (30.1%)	0.841
Fatigue	72 (86.7%)	194 (82.2%)	0.338
Myalgia/arthralgia	22 (26.5%)	61 (25.8%)	0.906
Dysphagia	4 (4.8%)	13 (5.5%)	0.809
COVID-19 treatment			
Antivirals	69 (83.1%)	201 (85.2%)	0.657
Corticosteroids	65 (78.3%)	19 (83.5%)	0.291
Antibiotics	70 (84.3%)	209 (88.6%)	0.317
Anticoagulant	61 (73.5%)	175 (74.2%)	0.906
Immune modulators	23 (27.7%)	62 (26.3%)	0.798
Thrombo-embolic complications			0.312
Yes	6 (8.5%)	11 (5.2%)	
No	65 (91.5%)	202 (94.8%)	
Clinical features			
Severe imaging features	29 (40.8%)	60 (28.2%)	0.046
Oxygen saturation on admission (<92%)	22 (31.0%)	54 (25.4%)	0.353
Respiratory rate on admission (>20/min)	38 (53.5%)	96 (45.1%)	0.216
Heart rate on admission (>100 bpm)	35 (66.0%)	123 (57.7%)	0.214

* Data reported as n (%) and calculated using the chi-square test and Fisher’s exact unless specified differently. BMI—body mass index; ICU—intensive care unit.

**Table 3:** Comparison of COVID-19 outcomes between patients of cases and controls admitted to the ICU.

Variables *	Cases (*n* = 71)	Controls (*n* = 213)	*p*-Value
Mean duration of hospital stay	18.2 ± 5.3	16.4 ± 6.1	0.027
Median duration from symptom onset until hospital admission	3.5 (4.0)	4.5 (6.5)	0.190
Viral clearance	16.8 ± 6.3	14.5 ± 6.8	0.012
Median duration from hospital admission to ICU admission	3.5 (5.5)	5.0 (7.0)	0.113
SOFA score	2.7 (3.1)	2.2 (3.4)	0.077
Median duration of ICU stay	7 (11)	5 (8)	<0.001
Severe in-hospital complications	17 (23.9%)	66 (31.0%)	0.258
Intubation	33 (46.5%)	104 (48.8%)	0.737
Mortality	19 (26.8%)	46 (21.6%)	0.369

* Data reported as n (%) and calculated using the chi-square test and Fisher’s exact unless specified differently. BMI—body mass index; ICU—intensive care unit.

**Table 4:** Comparison of cytokines and inflammatory markers at hospital admission between cases and controls.

Variables *	Normal Range	Cases (*n* = 71)	Controls (*n* = 213)	*p*-Value
Procalcitonin (ug/L)	0–0.25	0.66 (0.38)	0.69 (0.33)	0.629
D-dimers (ng/mL)	<250	262 (93)	268 (98)	0.551
IL-6 (pg/mL)	0.8–6.4	7.8 (3.9)	7.1 (3.7)	0.104
TNF-α (pg/mL)	7.8–12.2	12.5 (4.0)	12.6 (4.2)	0.590
Ferritin (ng/mL)	20–250	228 (72)	214 (66)	0.282
ESR (mm/h)	0–22	26 (20)	20 (18)	0.002
CRP (mg/dL)	0–10	24 (15)	17 (14)	<0.001
Fibrinogen (g/L)	2–4	4.1 (2.7)	4.8 (3.0)	0.088

* Data reported as median (IQR) and calculated using the Mann–Whitney U-test. CRP—C-reactive Protein; IL—interleukin; TNF—tumor necrosis factor; ESR—erythrocyte sedimentation rate.

**Table 5:** Comparison of cytokines and inflammatory markers at ICU admission between cases and controls.

Variables *	Normal Range	Cases (*n* = 71)	Controls (*n* = 213)	*p*-Value
Procalcitonin (ug/L)	0–0.25	0.93 (0.68)	0.82 (0.50)	0.063
D-dimers (ng/mL)	<250	318 (166)	304 (157)	0.292
IL-6 (pg/mL)	0.8–6.4	17 (9)	11 (10)	0.004
TNF-α (pg/mL)	7.8–12.2	15.0 (6.4)	14.9 (6.8)	0.527
Ferritin (ng/mL)	20–250	242 (92)	257 (72)	0.406
ESR (mm/h)	0–22	26 (20)	20 (18)	0.094
CRP (mg/dL)	0–10	27 (17)	21 (11)	<0.001
Fibrinogen (g/L)	2–4	6.1 (3.3)	5.8 (3.0)	0.215

* Data reported as median (IQR) and calculated using the Mann–Whitney U-test. CRP—C-reactive protein; IL—interleukin; TNF—tumor necrosis factor; ESR—erythrocyte sedimentation rate.

**Table 6:** Multivariate regression analysis in determining the influence of ethnicity for elevated inflammatory markers.

	General Population			Roma Ethnicity		
Constants (Dependent)	β	(95% CI of β)	*p*-Value	β	(95% CI of β)	*p*-Value
1× Normal Range						
CRP >10 mg/dL	1.39	0.94–1.82	0.334	1.58	0.99–1.93	0.217
ESR >22 mm/h	0.94	0.62–1.57	0.390	1.66	0.89–1.87	0.195
IL-6 >6.4 pg/mL	0.86	0.70–1.64	0.261	1.27	0.90–1.74	0.113
2× Normal Range						
CRP >20 mg/dL	1.53	0.98–2.42	0.066	1.93	1.15–3.66	0.020
ESR >44 mm/h	1.15	0.98–1.49	0.081	1.41	1.22–2.38	0.104
IL-6 >12.8 pg/mL	0.98	0.91–1.53	0.473	1.85	1.02–2.79	0.044

CI—confidence interval; β—risk estimate; CRP—C-reactive protein; ESR—erythrocyte sedimentation rate; IL—interleukin.

## Data Availability

Data are available on request.
